# Is there still a place for autologous salvage transplantation in relapsed/refractory multiple myeloma in the era of novel therapies?

**DOI:** 10.1007/s00277-025-06262-9

**Published:** 2025-02-26

**Authors:** Simone Karp, Karolin Trautmann-Grill, Paul Warncke, Dominik Zolnowski, Christoph Röllig, Marcel Pannach, Jessica Zinn, Frank Kroschinsky, Anke Morgner, Malte von Bonin, Annette Hänel, Regina Herbst, Stephan Fricke, Martin Bornhäuser, Mathias Hänel, Raphael Teipel

**Affiliations:** 1 Department of Internal Medicine III, Klinikum Chemnitz gGmbH, Chemnitz, Germany; 2 Department of Internal Medicine I, Faculty of Medicine, University Hospital Carl Gustav Carus, TUD Dresden University of Technology, Dresden, Germany; 3 Medical Campus Chemnitz, Faculty of Medicine, University Hospital Carl Gustav Carus, TUD Dresden University of Technology, Dresden, Germany

**Keywords:** Multiple myeloma, Relapse, Salvage autologous, Retransplantation, Autologous hematopoetic cell transplantation

## Abstract

For patients (pts) with relapsed or refractory multiple myeloma (RRMM) after previous autologous hematopoietic cell transplantation (AHCT), novel agents, cellular and immunotherapies are increasingly available. Options for second-line treatment mostly include triplet regimens based on proteasome inhibitors, immunomodulatory drugs and anti-CD38 monoclonal antibodies and since recently also CAR T cells. The importance of autologous salvage transplantation (retransplantation, Re-AHCT) has significantly decreased in recent years due to the availability of many new treatment options. Therefore, we performed a retrospective analysis of 171 pts cases with RRMM who received Re-AHCT between 2002 and 2021. With a median follow-up of 74.7 months, the 5-year rates of progression-free survival (PFS) and overall survival (OS) were 18% (median 20.6 months) and 57% (median 65.0 months), respectively, the 100-day mortality rate was 4%. Multivariate analysis identified R-ISS stage and duration of previous response (DoR) as independent prognostic factors for PFS and OS. While the revealed high-risk population (R-ISS stage II/III, DoR ≤ 24 months) was associated with a significantly worse PFS (HR 2.728) and OS (HR 3.129), the low-risk group (R-ISS I, DoR > 24 months) achieved a median PFS and OS of 45.0 months and 80.2 months, respectively. Therefore, Re-AHCT could remain an option in such prognostically favorable pts with RRMM even in the era of novel therapies especially when more potent treatment modalities are not available.

**Supplementary Information:**

The online version contains supplementary material available at 10.1007/s00277-025-06262-9.

## Introduction

The current standard of care in newly diagnosed multiple myeloma (NDMM) includes consolidation by high dose therapy (HDT) followed by autologous hematopoietic cell transplantation (AHCT) [1] for eligible patients (pts) up to the age of 65–70 years [2, 3]. However, the majority of these pts suffer a relapse or progression [4]. Second-line treatment mostly includes triplet regimens based on proteasome inhibitors (PIs) and/or immunomodulatory drugs (IMiDs) in combination with anti-CD38 monoclonal antibodies (mAbs) [5–10], steroids and/or classic cytotoxic chemotherapy. Starting with the approval of pomalidomide in 2013, many novel drugs and immunotherapies became available for salvage therapy of pts with relapsed/refractory multiple myeloma (RRMM).

In allogeneic HCT, the advantage of lower relapse rates is counterbalanced by the risk of increased transplant-related mortality (TRM) [11]. Therefore, the number of allogeneic HCT in RRMM has declined in recent years due to the development of new treatment approaches.

Bispecific T-cell engagers directed against B-cell maturation antigen (BCMA) or GPRC5D on myeloma cells and CD3 on T lymphocytes [12–14], as well as anti-BCMA chimeric antigen receptor (CAR) T cell therapies [15] and antibody-drug conjugates (ADC) [16] have been developed in rapid succession for the treatment of RRMM.

In addition, several studies have shown that in selected pts with RRMM, autologous salvage transplantation (retransplantation, Re-AHCT) may also be a potential option, associated with low TRM and long-term remissions [17–30]. However, several factors such as patient characteristics, outcome, response rates and treatment characteristics (maintenance and number of pts treated with novel drugs) differ between studies and justify the need for long-term evaluations.

The objective of our study was to assess the long-term outcome of pts with RRMM who underwent HDT/Re-AHCT. In addition, the impact of potential prognostic factors on progression-free survival (PFS) and overall survival (OS) should be investigated to identify pts who may continue to benefit from Re-AHCT, especially in the context of novel treatment options.

## Materials and methods

### Study design and patient cohort

The medical records of pts at Klinikum Chemnitz and University Hospital Dresden with RRMM at first or subsequent relapse or disease progression after initial HDT/AHCT who received a second or third HDT/AHCT as part of a salvage treatment were screened and retrospectively reviewed. All data were collected from each patient’s medical records, including demographic data, disease and treatment characteristics, as well as laboratory, pathology and cytogenetic test results at the time point of the Re-AHCT. The analysis included all pts cases who underwent Re-AHCT at the study centers between June 2002 and October 2021. During this time, we identified 171 Re-AHCTs performed in 168 different pts. Pts with Re-AHCT as part of a tandem transplantation were included in the analysis. Moreover, pts with a second Re-AHCT (*n* = 3) were considered in the patient cohort as individual pts cases. The patient data was pseudonymized by assigning a unique patient number. For statistical analysis, the data was then anonymized. Missing data was considered to be missing completely at random and not adjusted for analysis. Pts who received no reinduction therapy or Re-AHCT followed by allogeneic HCT were not included. Pts with loss to follow-up (*n* = 10) were censored at the last visit or at the end of the study period (death). The cut-off date for data was March 31st, 2023. The study was approved by the regional ethics committees of the State Chamber of Physicians of Saxony (EK-BR-40/23 − 1).

### Definitions and outcome assessment

A transplant was defined as Re-AHCT if the patient had relapsed after previous HDT/AHCT (either single or tandem) and subsequently received another AHCT as part of salvage therapy, regardless of how many treatments they had received after the initial HDT/AHCT. If Re-AHCT was given as part of an autologous tandem transplant, only the second transplantation of the tandem Re-AHCT was analyzed. For pts with a second independent Re-AHCT, OS for the first AHCT was censored at the time of the second Re-AHCT. Pretreatment includes all treatments from diagnosis of multiple myeloma until the current relapse/disease progression. Salvage therapy was defined as reinduction treatment prior to Re-AHCT.

Remission status was assessed according to International Myeloma Working Group (IMWG) criteria [31, 32]. Patients were assigned to the R-ISS groups I and II/III based on the available data for beta-2-microglobulin, albumin, HRCA [del(17p); t(4;14), t(14;16)] and LDH or based on the stage determined from the medical records. Response to salvage therapy at the time of Re-AHCT was defined as follows: sensitive relapse (≥partial remission (PR) after the first-line salvage regimen), refractory relapse (≥PR after ≥2 different salvage regimens), or total refractory (never achieved ≥ PR after salvage therapy). The duration of previous response (DoR) after previous AHCT was calculated from the day of the stem cell infusion of the previous AHCT until the date of relapse or disease progression. Toxicity grading was performed according to the Common Terminology Criteria of Adverse Event (CTCAE) version 5.0.

Primary endpoints were PFS and OS. PFS was defined as the time from the date of Re-AHCT to disease progression, relapse or death, whereas OS was defined as the time from the date of Re-AHCT to the date of death from any cause. For pts who received a second Re-AHCT (*n* = 3) at a later relapse after previous Re-AHCT, OS after first Re-AHCT was censored at the date of second Re-AHCT.

### Statistical analysis

Kaplan-Meier estimator was used to determine the median OS and PFS. Inverse Kaplan-Meier method was used to determine the median follow-up time. Survival curves were compared using two-sided log rank test. *P* value of 0.05 was considered as statistically significant. Uni- and multivariate Cox proportional hazards regression analyses were performed for the variables sex (male vs. female), age (≤65 years vs. >65 years), soft-tissue plasmacytoma (STP; no vs. yes), number of previous AHCT (1 vs. >1), number of relapses (1 vs. >1), R-ISS stage (I vs. II + III), serum lactate dehydrogenase (LDH; normal vs. elevated), DoR (> 24 months vs. ≤24 months), high-risk cytogenetic aberrations (HRCA - shown both exclusively according to R-ISS as del17p13, t(4;14), t(14;16) and as HRCA (R-ISS) and/or t(14;20) and/or gain/amp 1q21), paraprotein type (IgA vs. others), response to first salvage therapy (yes vs. no), conditioning regimen (high-dose melphalan/HD-Mel vs. busulfan-melphalan/Bu-Mel), previous systemic therapy (classical chemotherapy vs. novel drugs), number of novel drug classes in salvage therapy (1 vs. >1), number of pretreatment regimens (≤2 vs. >2), lenalidomide maintenance therapy (no vs. yes) and time of Re-AHCT (2002–2013 vs. 2014–2021). Multivariate Cox proportional hazards regression model was built based on univariate analysis with *P* < 0.1 as entry criterion (complete model) and by stepwise reduction using *P* > 0.1 as exclusion criterion (reduced model). Data analysis and visualization was performed using IBM SPSS^Ⓡ^ 25 and GraphPad Prism (v.6.0).

## Results

### Patients

Based on the eligibility criteria, we identified 171 Re-AHCTs in 168 RRMM pts between June 2002 and October 2021. Clinical and disease-specific characteristics for all pts are shown in Table 1. The median age was 64 years (range: 39–76), with 68 pts (40%) being over 65 years old at the time of Re-AHCT. Almost two thirds of pts were male. Thirty-eight pts (22%) had a primary (*n* = 24) or secondary (*n* = 14) STP. Twenty-nine pts had only a paraskeletal STP, and 9 pts were found to have extramedullary organ involvement.

More than two thirds (69%) of the pts had received at least one of the novel classes of IMiD, PI or anti-CD38 mAb prior to their first AHCT (pretreatment) and almost all pts (99%) received at least one of the novel agents in salvage therapy prior to Re-AHCT. All pts received reinduction therapy before Re-AHCT. Forty-eight pts (28%) did not respond to the first-line salvage regimen. Of these, 27 pts responded to the second-line salvage regimen. The remaining 21 pts did not achieve PR before Re-AHCT. Lenalidomide maintenance therapy was applied in 19 (11%) pts after the previous AHCT.

HDT was performed with HD-Mel in 154 pts (90%). Other conditioning regimens were Bu/Mel (*n* = 13), BEAM (BCNU, etoposide, cytarabine, melphalan; *n* = 1), busulfan/thiotepa (*n* = 1) and high dose bendamustine (*n* = 2). For Re-AHCT, either sufficient cryopreserved autologous stem cells were still available, or another harvest of CD34-positive cells was performed during the salvage therapy.

**Table 1:** Patient characteristics

Characteristics at Re-AHCT		Number of Patient cases (*n* = 171)	%	Median (Range)
Age (years)				64 (39–76)
Sex	female	59	35%	
	male	112	65%	
ECOG PS	0–1	168	98%	
	≥ 2	3	2%	
Heavy/light chain isotype	IgG	93	54%	
	IgA	43	25%	
	IgD	5	3%	
	Kappa-LC	17	10%	
	Lambda-LC	11	7%	
	Asecretory	2	1%	
R-ISS stage ^1^	R-ISS I	69	41%	
	≥ R-ISS II	98	57%	
	Not available	4	2%	
HRCA (R-ISS) ^2^	Yes	21	12%	
	No	116	68%	
	Not available	34	20%	
HRCA (R-ISS) and/or t(14;20) and/or gain/amp 1q21^3^	Yes	52	30%	
	No	46	27%	
	Not available	73	43%	
Serum-LDH	Normal	91	53%	
	Elevated	69	41%	
	Not available	11	6%	
Soft-tissue plasmacytoma		38	22%	
Disease status	1st relapse	146	86%	
	2nd relapse	19	11%	
	3rd relapse	4	2%	
	> 3 relapses	2	1%	
DoR after previous AHCT	≤ 12 months	27	16%	
	13–24 months	48	28%	
	25–36 months	31	18%	
	> 36 months	65	38%	
	Median DoR (months)			30 (3-155)
Pretreatment	No. of regimens			2 (2–6)
	0 novel drug class ^4^	53	31%	
	1 novel drug class ^4^	102	60%	
	2 novel drug classes ^4^	16	9%	
	1 previous AHCT	142	83%	
	2 previous AHCT	26	15%	
	Previous Re-AHCT	3	2%	
Lenalidomide maintenance after initial AHCT		19	11%	
Salvage therapy	No. of regimens			1 (1–10)
	0 novel drug class ^4^	2	1%	
	1 novel drug class ^4^	80	47%	
	2 novel drug classes ^4^	83	49%	
	3 novel drug classes ^4^	6	3%	
Response to salvage therapy	Sensitive relapse	123	72%	
	Refractory relapse	27	16%	
	Total refractory	21	12%	
Remission status at Re-AHCT	CR	42	25%	
	VGPR	59	34%	
	PR	49	29%	
	< PR	21	12%	
Conditioning regimen	HD-Mel	154	90%	
	Bu-Mel	13	8%	
	Others	4	2%	
CD34 + cells, x 10^6^/kg				3.5 (1.1–18.1)
Time of Re-AHCT	2002–2013	71	42%	
	2014–2021	100	58%	
Allogeneic HCT at later stage ^5^		29	17%	

^1^ R-ISS based on laboratory data or medical records. ^2^ HRCA (R-ISS) include del17p13, t(4;14), t(14;16). ^3^ HRCA include HRCA (R-ISS) and/or t(14;20) and/or gain/amp 1q21. ^4^ Novel drugs include PI, IMiD and anti-CD38 mAb. ^5^ Including 3 pts who received allogeneic HCT due to secondary malignancy. Abbreviations: (A)HCT: (autologous) hematopoietic cell transplantation; CD: cluster of differentiation; mAb: monoclonal antibody; CR: complete remission; ECOG PS: Eastern Cooperative Oncology Group Performance Status; HRCA: high-risk cytogenetic aberration; Ig: Immunoglobulin; IMiD: immunomodulatory drug; LC: light chain; PI: proteasome inhibitor; PR: partial remission; R-ISS: Revised International Staging System; VGPR: very good partial remission

### Outcome and survival

Response assessment (*n* = 154) 3 months after Re-AHCT showed complete remission (CR) in 70 pts (41%), very good partial remission (VGPR) in 41 pts (24%) and PR in 24 pts (14%), corresponding to an overall response rate (ORR) of 79%. Twelve pts (7%) did not achieve at least PR, including 8 pts (5%) with progressive disease. Seven pts (4%) died within 100 days of Re-AHCT due to infection (3 pts), refractory/progressive multiple myeloma (2 pts), or of unknown causes (2 pts). In 17 pts (10%), no data were available to assess the remission status.

**Fig. 1:**
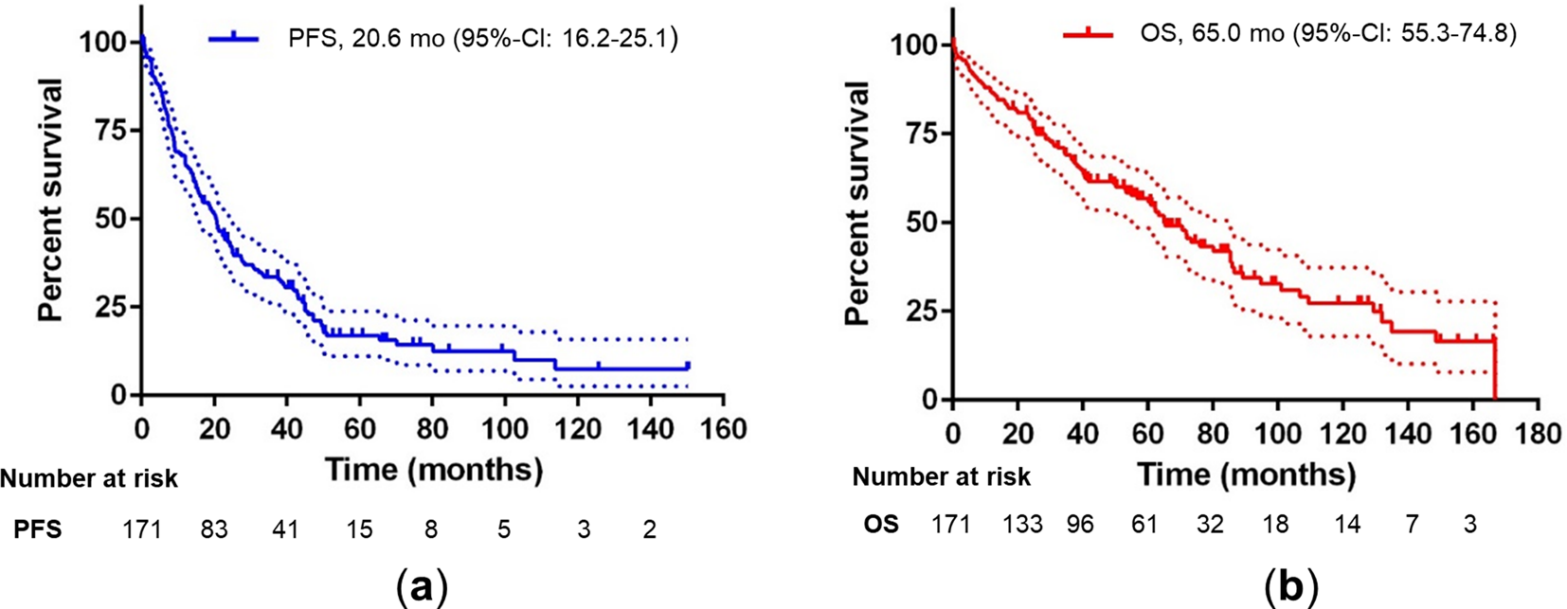
Progression-free survival (**a**) and overall survival (**b**) for 171 patient cases after Re-AHCT

With a median follow-up of 74.7 months, the 5-year rates of PFS and OS after Re-AHCT were 18% (median 20.6 months; 95%-CI: 16.2–25.1) and 57% (median 65.0 months; 95%-CI: 55.3–74.8), respectively (see Fig. 1).

As expected, response to salvage therapy (≥ PR) was associated with significantly improved PFS and OS (see Figure S1). In addition, pts with a sensitive relapse who achieved remission after first-line salvage regimen (*n* = 123) showed a significantly (*P* < 0.05) improved PFS and OS, compared to total refractory pts (*n* = 21). Pts with sensitive relapse showed improved PFS (25.1 months vs. 14.4 months; *P* = 0.025) but not OS (80.2 months vs. 72.1 months, *P* = 0.539) compared to pts with refractory relapse (*n* = 27). The respective survival curves and the impact of different remission statuses (CR, VGPR, PR or < PR) on PFS and OS after first or subsequent salvage therapy is shown in Figure S2.

### Toxicity

Toxicity data are shown in Table 2. More than half of the pts suffered non-hematological toxicities ≥ grade 3, mainly infections (38%). Sinusoidal obstruction syndrome (SOS) occurred in 4 of 13 pts who received busulfan (16 mg/kg p.o.) and melphalan (100 mg/m^2^ IV) as conditioning. Engraftment syndrome, defined as a high and well-tolerated fever of non-infectious origin at the time of the appearance of the first neutrophils in the peripheral blood, often accompanied by skin rash and diarrhea [33], was seen in almost one-fifth of our pts. In one patient, acute skin GvHD (grade 1) was histologically confirmed, which responded to steroid treatment.

**Table 2:** Toxicity after Re-AHCT

Characteristics		Number of patient cases (*n* = 171)	%
Toxicities (grade ≥ 3)	Hematological	170	99%
	Non-hematological	97	57%
	Infection	65	38%
	Engraftment syndrome	30	18%
	SOS after busulfan (*n* = 13)	4	31%
	Hemorrhages	2	1%
	Acute GvHD (skin)	1	1%
Day 100 mortality ^1^		7	4%
SPM ^2^		17 ^3^	10%
	MDS/AML	9	5%
	ALL	1	1%
	Solid cancer ^4^	9	5%

^1^ Data available for 166 pts. ^2^ Data available for 169 pts; ^3^ 2 pts MDS and skin cancer, ^4^ 2 prostate, 1 lung, 1 thyroid, 1 malignant melanoma, 3 cutaneous squamous cell carcinoma, 1 pancreatic cancer. Abbreviations: ALL: acute lymphoblastic leukemia, AML: acute myeloid leukemia, GvHD: graft-versus-host disease, MDS: myelodysplastic syndrome, SOS: sinusoidal obstruction syndrome, SPM: Second primary malignancy

### Prognostic factors and risk score

Various prognostic variables were examined for their impact on PFS and OS in a univariate and multivariate Cox proportional hazards regression analysis. The detailed results of the univariate analysis can be found in the supplement (Table S1).

Based on the univariate analysis, a multivariate Cox proportional hazards regression analysis was performed (results of complete model are shown in Table S2). After subsequent stepwise reduction according to the predefined criteria, the analysis confirmed DoR after previous AHCT ≤24 months, R-ISS stage II or III and remission status at Re-AHCT (< PR) as potential risk factors for both PFS and OS (see Table 3).

**Table 3:** Multivariate Cox proportional hazards regression analysis after stepwise reduction (reduced model). Hazard ratio (HR) and 95% confidence interval (CI) for PFS and OS

Variable	HR_PFS_	95% CI	*P* value	HR_OS_	95%-CI	*P* value
DoR ≤ 24 months	1.887	1.310–2.718	0.001	1.903	1.252–2.894	0.003
R-ISS stage II + III	1.631	1.106–2.405	0.014	1.536	0.988–2.390	0.057
< PR at Re-AHCT	3.691	2.222–6.130	< 0.001	3.439	2.073–5.707	< 0.001
Pretreatment > 2 regimens	1.976	1.294–3.017	0.002	-	-	-
STP	1.970	1.292–3.003	0.002	-	-	-

Abbreviations: DoR: duration of previous response, PR: Partial remission, Re-AHCT: autologous retransplantation, R-ISS: Revised International Staging System, STP: Soft-tissue plasmacytoma

Since most of the 21 pts with < PR were in the high-risk group (*n* = 15), the stratification has only a minor effect on the prognostically favorable (*n* = 1) and intermediate-risk (*n* = 5) pts. Therefore < PR was not included as a separate factor in the stratification model. For the stratification model, pts with R-ISS I and DoR > 24 months were classified as low-risk, whereas pts with R-ISS II or III were classified as intermediate-risk (DoR > 24 months) or high-risk (DoR ≤ 24 months) as shown in Table 4.

**Table 4:** Prognostic stratification based on R-ISS and duration of response (DoR)

	*R*-ISS^1^	DoR	Patient cases	Median PFS (95%-CI), months	HR PFS (*P* value)	Median OS (95%-CI), months	HR OS (*P* value)
Low risk	I	> 24 months	43	44.98 (24.72–65.23)	1.000	80.19 (60.02-100.36)	1.000
Intermediate risk	I	≤ 24 months	77	20.98 (16.82–25.14)	1.857 (0.007)	85.41 (67.54-103.29)	1.416 (0.253)
	II/III	> 24 months					
High risk	II/III	≤ 24 months	47	9.04 (6.45–11.63)	2.728 (< 0.001)	23.64 (7.72–39.56)	3.129 (< 0.001)

^1^ data available for 167 patients

**Fig. 2:**
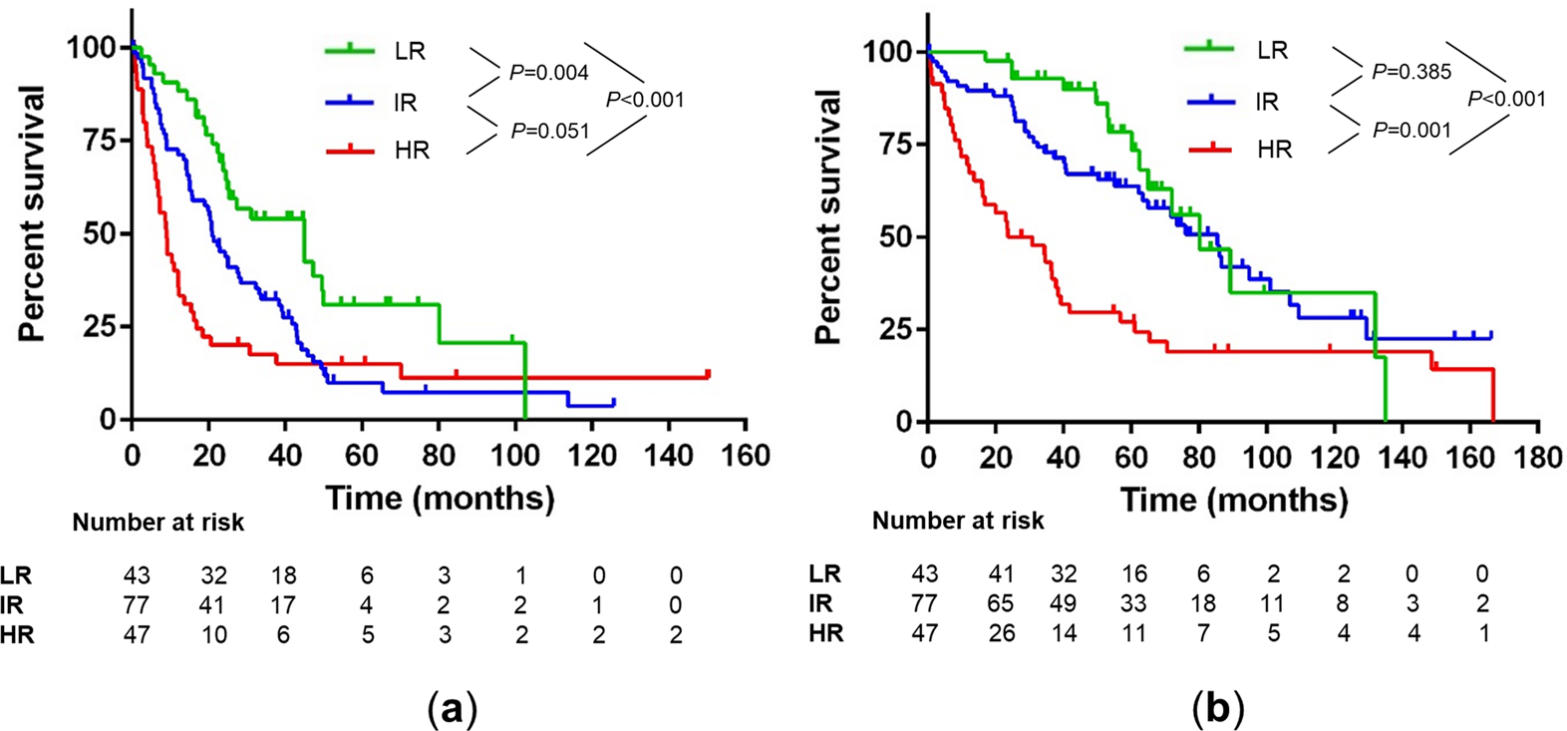
Progression-free survival (**a**) and overall survival (**b**) by risk groups. Low risk (LR), intermediate risk (IR) and high risk (HR) based on R-ISS and duration of response for 167 patient cases

The majority of the pts belonged to the intermediate-risk group (46%), whereas 26% and 28% were in the low and high-risk group, respectively. The high-risk population showed an approximately threefold increased risk for both PFS and OS (see Fig. 2). In addition, the subgroup of chemosensitive patients (> PR) showed comparable results to the entire study population when using our risk stratification (see Figure S3).

## Discussion

Although consolidation by HDT/AHCT is an established standard in first-line therapy for NDMM pts up to approximately 65–70 years of age, the importance of Re-AHCT in pts with RRMM has significantly decreased in recent years. In first or second relapse, triplet regimens, based on a PI (bortezomib, carfilzomib, ixazomib) and/or an IMiD (lenalidomide, pomalidomide) in combination with an anti-CD38 mAb (daratumumab, isatuximab) are regularly used and have shown convincing data [5–8, 10, 34–46].

Re-AHCT is an option in RRMM to consolidate a remission achieved after salvage therapy. Table 5 provides an overview of published retrospective and prospective analyses on Re-AHCT. In the last decades only 2 prospective trials [23, 26] investigated Re-AHCT for RRMM. Cook et al. [23] compared salvage AHCT with weekly oral cyclophosphamide for 12 weeks, which from today’s perspective, was not a potent comparative study arm. Recently, the long-term follow-up results of the GMMG-ReLApsE trial were presented, which describe no survival benefit in favor of Re-AHCT for both PFS (19.3 months vs. 20.5 months, *P* = 0.9; HR 0.98) and OS (62.7 months vs. 67.1 months, *P* = 0.44; HR 0.89) compared to a continuous lenalidomide-based regimen. In the multivariate analysis, only DoR > 48 months after frontline transplant proved to be a positive prognostic factor for survival both for Re-AHCT and for continuous lenalidomide/dexamethasone.

**Table 5:** Results of retrospective and prospective studies on Re-AHCT

Study	No. of pat-ients	Early relapse ≤ 18 mo	Median DoR (mo)	High risk cytogenetics	Refractory (< PR at Re-AHCT)	Day 100 mortality	Median PFS (mo)	Median OS (mo)	Median FU (mo)	ORR	5-yr OS	IMiD Main-tenance^1^	Pre-treatment with novel drugs
Olin et al., 2009 [17]	41	N/A	21	N/A	63%	7%	8.5	20.7	15	55%	∼13%	44% (T)	46% (T) 22% (L) 59% (B)
Jimenez-Zepeda et al., 2012 [18]	81	N/A	39.0	N/A	14%	2.6%	16.4	53.0	36	97.4%	N/A	N/A	27% (B, L, T)
Sellner et al., 2013 [19]	200	26.5%	64.6	N/A	42%	3.0%	15.2	42.3	57	80.4%	N/A	12% (T or B)	7 (B) 22% (L) 38% (T)
Michaelis et al., 2013 [20]	187	N/A	18	N/A	60%	2% (1-yr NRM)	∼12.0	∼33.0	47	68%	29%	N/A	N/A
Singh Abbi et al., 2015 [21]	75	16% (< 12 mo)	21.9	19%	43%	5.3%	10.1	22.7	33	77%	∼30%	N/A	90% (PI or IMiD)
Grövdal et al., 2015 [22]	111	N/A	28.8	N/A	37%	N/A	28.8	48.0	64	N/A	N/A	N/A	36% (B) 13% (T) 7% (L), 7% (PI + IMiD)
Cook et al., 2016 [23]	89	3%	31.2	16%	N/A	1%	19.0	67.0	50	N/A	∼60%	17% (T)	100% (B)
Garderet et al., 2018 [24]	482	36%	24.0	N/A	46%	4%	13.0	33.0	62	82%	∼30%	N/A	N/A
Dhakal et al., 2021 [25]	975	N/A	N/A	N/A	18%	1%	∼12.0	NR	35	N/A	68% (3-yr)	N/A	N/A
Goldschmidt et al., 2021 [26]	98	N/A	N/A	43%	40%	0%	20.7	NR	36.8	82%	71.8% (3-yr)	N/A	77% (B) 22% (T) 9% (L)
Drozd-Sokołowska et al., 2022 [27]	305	N/A	30.6	N/A	31%	1%	17.0	51.0	31	N/A	52% (4-yr)	N/A	N/A
Baertsch et al., 2023 [28]	98	N/A	N/A	43%	40%	N/A	20.5	67.1	99	N/A	N/A	N/A	77% (B) 22% (T) 9% (L)
Tilmont et al., 2023 [29]	51	N/A	N/A	29%	4%	4%	29.5	NR	36.7	100%	85% (3-yr)	10% (L), 4% (T)	14% (L)
Sauer et al., 2024 [30]	171	N/A	47.0	N/A	13%	N/A	29.0	NR	33	96%	N/A	17% (L), 7% (L)	91% (B) 55% (L)

^1^ maintenance therapy after previous AHCT – drugs; B = bortezomib; L = lenalidomide, T = thalidomide. Abbreviations: N/A: not available, mo = months; yr = years

The observed PFS (20.6 months) and OS (65.0 months) in our analysis were comparable to the two prospective studies [23, 26, 28] in this period (see Table 5). Since the earlier retrospective studies date from a time before the approval of PI and IMiD, this could explain the poorer results from this time. The majority of pts in our study cohort received Re-AHCT at first relapse (86%) and showed a comparatively good response to salvage treatment before Re-AHCT (only 12% < PR), compared to most of the studies mentioned in Table 5. Limited availability of data regarding treatment with novel agents or the proportion of pts with maintenance therapy limits the comparability of these studies.

In addition to response to salvage therapy, multivariate analysis indicated DoR after previous AHCT as a predictive factor for the outcome after Re-AHCT. Various studies reported DoR between ≥18 months after initial AHCT [19, 24, 47] and ≥36 months after AHCT with lenalidomide maintenance [48]. In our study, a DoR ≤ 24 months and an R-ISS stage II or III were selected for risk stratification, as both proved to be independent risk factors for PFS and OS in the multivariate analysis. Compared to the score of Sellner et al. [19], our prognostic stratification takes into account the R-ISS (including HRCA and serum LDH) and a longer prior DoR (due to improved first-line therapy). A low-risk group (R-ISS I, DoR > 24 months) could be identified. For these pts with a median PFS of 45.0 months and OS of 80.2 months, Re-AHCT still appears to be a considerable treatment option, despite the lack of randomized comparative data for the use of continuous triplet-combination therapies in this low-risk subgroup. Of particular note in these low-risk pts is the long-term treatment-free interval following Re-AHCT, which is not achieved in pts who are continuously treated with salvage therapy. Furthermore, the possible cost effectiveness of a salvage transplantation compared to modern continuous triplet regimens or even a CAR-T-cell therapy needs to be considered. Nevertheless, the risk score needs to be validated in larger cohorts, especially considering the current role of maintenance therapy.

The toxicity related to the Re-AHCT in our study was tolerable with mainly hematological and infectious complications as expected. Despite the inclusion of pts up to 76 years of age, the early TRM after Re-AHCT was low (3 pts died of infectious complications, 2 pts of unknown cause), which reassures the safety of the procedure even in the relapsed setting. In addition, we did not observe an increased number of second primary malignancies (SPM) compared to published data [25, 26].

In the age of cell and immunotherapies, our data must be discussed in the context of those modern therapy options. In the recently published KarMMa-3 trial [49], intensively pretreated pts who received CAR T cell therapy with ide-cel as third-line therapy (or later) achieved a median PFS of 13.3 months with an ORR of 71% compared to standard of care triplet therapies. Real world data [50] confirmed the efficacy lately, reporting a median PFS and OS of 8.5 and 12.5 months, respectively. Nevertheless, these pts have been extensively pretreated with a relevant proportion of triple-class refractory pts at the time of trial entry. In the CARTITUDE-4 study [51], cilta-cel (at least second-line therapy) showed tremendous efficacy of CAR T cell therapy in early lines of therapy and is now considered the most potent treatment option in second line. However, these treatment options are currently still limited in wide parts of the world by accessibility, logistical efforts and costs, and are therefore not a general option for all pts. Other approved targeted therapies for triple-class exposed RRMM include bispecific antibodies against BCMA (teclistamab, elranatamab) [12, 14, 52–58] or GPRC5D (talquetamab) [13, 59]. These agents are currently also being extensively evaluated in earlier lines of therapy compared to standard salvage therapies.

The limitations of our study are the retrospective data analysis and the heterogeneity of the patient population. A proportion of pts in our dataset were treated at a time when modern triplet combinations were not widely used and maintenance therapy after first-line AHCT was no standard. The low proportion of lenalidomide maintenance therapy in our study population limits the comparability of the study. Finally, the currently increasing availability and use of highly potent cellular immunotherapies in earlier lines must be taken into consideration when interpreting our data.

## Conclusions

Despite the limitations of a retrospective analysis, we conclude that Re-AHCT could remain an option for prognostically favorable pts with chemosensitive late relapse, even in the era of modern cell and immunotherapies, especially when more potent options like CAR-T-cell treatment are not broadly available. In the case of response to modern salvage therapy (including novel drugs), Re-AHCT offers selected pts the chance of a renewed long-term remission with a manageable toxicity profile and the benefit of a long-term intensive treatment-free interval.

## Electronic supplementary material

Below is the link to the electronic supplementary material.

Supplementary Material 1

## Data Availability

No datasets were generated or analysed during the current study.
