# Supplementary material for: Is there still a place for autologous salvage transplantation in relapsed/refractory multiple myeloma in the era of novel therapies?
Source: Ann Hematol. 2025 Feb 26;104(3):1735–45. doi: 10.1007/s00277-025-06262-9 (PMC12031966; doi:10.1007/s00277-025-06262-9)
Supplement: Supplementary file 1 — Supplementary Material 1 [file 277_2025_6262_MOESM1_ESM.pdf]

Supplementary Information

Is there still a place for autologous salvage transplantation in relapsed/refractory multiple myeloma in the era of novel therapies?

Journal

Annals of Hematology

Authors

Simone Karp[1,3], Karolin Trautmann-Grill[2], Paul Warncke[1,3], Dominik Zolnowski[1,3], Christoph Röllig[2], Marcel Pannach[1], Jessica Zinn[1], Frank Kroschinsky[2], Anke Morgner[1,3], Malte von Bonin[2], Annette Hänel[1], Regina Herbst[1,3], Stephan Fricke[1,3], Martin Bornhäuser[2], Mathias Hänel[1,3] and Raphael Teipel[2]

Affiliations

[1] Department of Internal Medicine III, Klinikum Chemnitz gGmbH, Chemnitz, Germany

[2] Department of Internal Medicine I, Faculty of Medicine and University Hospital Carl Gustav Carus, TUD Dresden University of Technology, Dresden, Germany

[3] Medical Campus Chemnitz, Faculty of Medicine and University Hospital Carl Gustav Carus, TUD Dresden University of Technology, Dresden, Germany

Correspondence: m.haenel@skc.de (Mathias Hänel)

Figures:

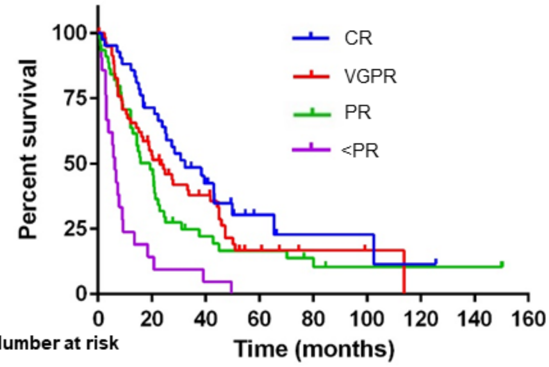

Number at risk

**CR**	42	29	14	4	2	2	1	0
**VGPR**	59	30	18	5	2	1	0	0
**PR**	49	21	8	6	4	2	2	2
**<PR**	21	3	1	0	0	0	0	0

(**a**)

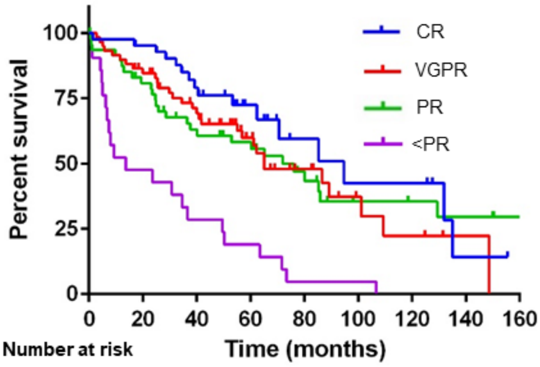

Number at risk

**CR**	42	39	28	13	7	5	5	1	0
**VGPR**	59	47	36	21	11	5	3	1	0
**PR**	49	37	27	23	13	7	6	5	3
**<PR**	21	10	6	4	1	1	0	0	0

(**b**)

Factor 1	Factor 2	P_{PFS}	P_{OS}
CR	VGPR	0.204	0.129
CR	PR	**0.024**	0.257
CR	<PR	**<0.001**	**<0.001**
VGPR	PR	0.320	0.836
VGPR	<PR	**<0.001**	**<0.001**
PR	<PR	**0.001**	**<0.001**

(**c**)

Fig. S1 Progression-free survival (PFS, a) and overall survival (OS, b) stratified by remission status at Re-AHCT. *P* values (two-sided log rank test) for PFS and OS comparing factor 1 vs. factor 2 (c).

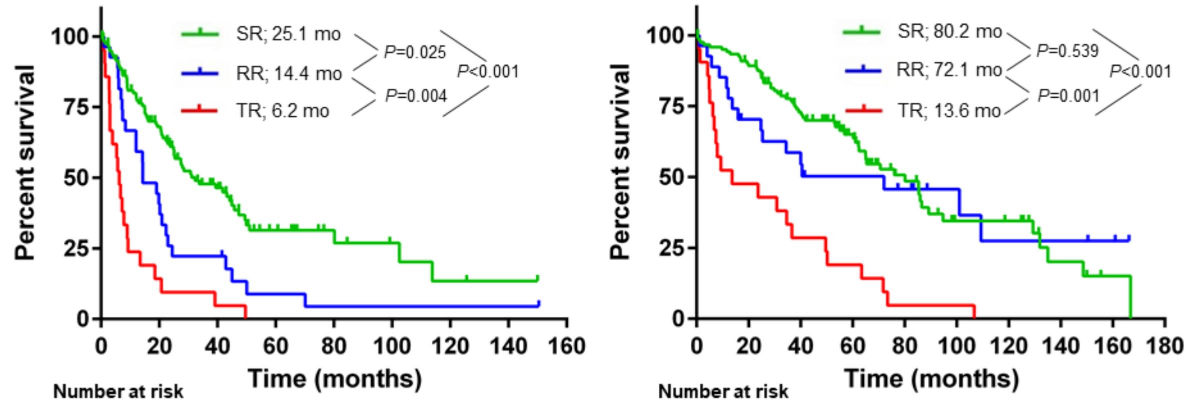

Number at risk

SR	123	69	34	13	7	4	2	1
RR	27	11	6	2	1	1	1	1
TR	21	3	1	0	0	0	0	0

(a)

Number at risk

SR	123	115	76	46	23	12	11	4	1
RR	27	18	14	11	8	5	3	3	2
TR	21	10	6	4	1	1	0	0	0

(b)

Response to salvage therapy	Remission status at Re-AHCT	Patient cases	Median PFS (95%-CI, mo)	Median OS (95%-CI, mo)
Sensitive Relapse	CR	40	38.4 (23.9-52.9)	94.8 (59.7-129.8)
	VGPR	46	28.0 (15.2-40.8)	65.0 (36.0-94.1)
	PR	37	20.5 (13.4-27.6)	65.3 (31.3-99.4)
Refractory Relapse	CR	2	14.2 (-)	40.5 (-)
	VGPR	13	19.1 (4.3-33.9)	34.5 (3.5-65.4)
	PR	12	14.3 (10.4-18.2)	not reached
Total Refractory	<PR	21	6.2 (4.3-8.2)	13.6 (0.0-37.0)

(c)

Fig. S2 Progression-free survival (PFS, a) and overall survival (OS, b) and median survival in months (mo) of 171 patient cases stratified by response to salvage therapy (Sensitive relapse – SR; Refractory Relapse – RR; Total Refractory – TR). Impact of response to salvage therapy and remission status at Re-AHCT on median progression-free survival (PFS) and overall survival (OS) is shown in (c).

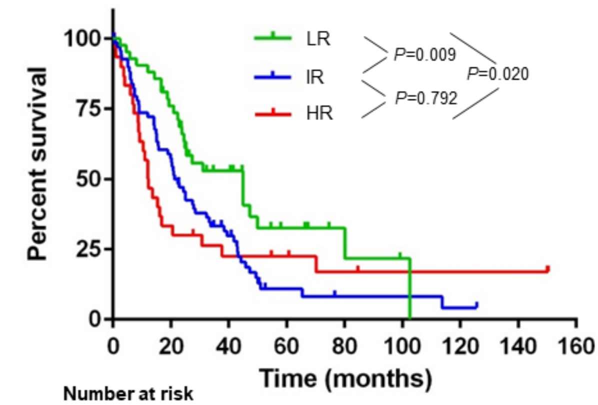

Number at risk

**LR**	42	31	17	6	3	1	0	0
**IR**	72	39	17	4	2	2	1	0
**HR**	32	10	6	5	3	2	2	2

(**a**)

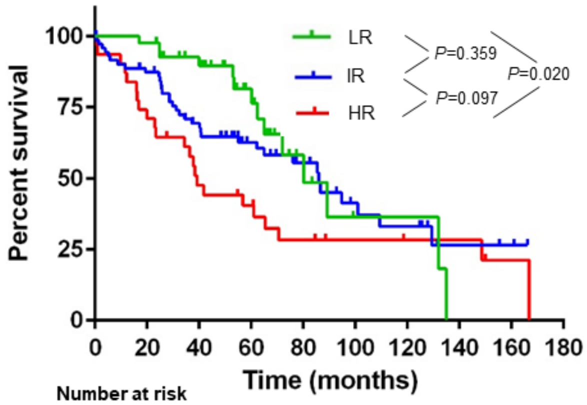

Number at risk

**LR**	42	40	31	16	6	2	2	0	0
**IR**	72	60	44	29	17	10	8	3	2
**HR**	32	22	14	11	7	5	4	4	1

(**b**)

	**R-ISS[1]**	**DoR**	**Patient cases**	**Median PFS (95%-CI), months**	**HR PFS (*P* value)**	**Median OS (95%-CI), months**	**HR OS (*P* value)**
Low risk	I	> 24 months	42	44.98 (18.04-71.91)	1.000	80.19 (60.25-100.13)	1.000
Intermediate risk	I	≤ 24 months	72	22.39 (17.54-27.24)	1.814 (0.012)	85.94 (66.85-105.03)	1.414 (0.276)
	II/III	> 24 months					
High risk	II/III	≤ 24 months	32	12.10 (8.57-15.63)	1.895 (0.025)	39.29 (32.2-46.36)	2.158 (0.027)

[1]data available for 146 patients

(**c**)

Fig. S3 Progression-free survival (PFS, a) and overall survival (OS, b) of chemosensitive patient cases (n=146; >PR) stratified by risk based on R-ISS and duration of response (DoR).Stratification, Median PFS and OS, as well as Hazard Ratios are provided in (c).

Tables:

Table S1 Univariate Cox proportional hazards regression analysis shows estimated median PFS (a) and OS (b) and corresponding hazard ratios (HR).

(a) – PFS

Variable	Factor	n	Median PFS (mo)	95% CI	HR	95% CI	*P* value
Sex	male	112	20.98	17.51-24.44	1.00		
	female	59	18.51	7.96-29.06	1.127	0.787-1.616	0.513
Age	≤65 years	105	15.49	13.23-17.73	1.00		
	>65 years	66	32.32	17.89-46.75	0.528	0.366-0.763	0.001
Previous AHCT	1	142	21.30	15.28-27.33	1.00		
	>1	29	19.86	12.23-27.49	1.297	0.854-1.969	0.223
Soft-tissue plasmacytoma	no	133	21.30	17.26-25.35	1.00		
	yes	38	15.88	11.14-20.62	1.544	1.031-2.310	0.035
Disease status	1 relapse	146	20.81	16.42-25.20	1.00		
	>1 relapse	25	15.65	10.34-20.96	1.541	0.980-2.421	0.061
DoR	>24 months	96	27.49	19.77-35.20	1.00		
	≤24 months	75	10.46	7.41-13.50	1.942	1.376-2.741	<0.001
R-ISS	I	69	27.45	18.20-36.71	1.00		
	II + III	98	15.49	9.40-21.57	1.534	1.076-2.187	0.018
LDH	normal	91	24.53	18.17-3.88	1.00		
	elevated	69	16.90	10.63-23.17	1.290	0.902-1.845	0.164
HRCA (R-ISS)	no	116	22.92	18.80-27.03	1.00		
	yes	21	15.09	10.27-19.91	1.411	0.846-2.353	0.187
HRCA (R-ISS) and/or t(14;20) and/or gain/amp1q21	no	46	22.95	2.06-43.84	1.00		
	yes	52	19.10	12.06-26.15	1.415	0.876-2.288	0.156
Paraprotein type	others	128	19.10	14.31-23.90	1.00		
	IgA	43	28.50	13.24-43.77	0.657	0.438-0.985	0.042
Response to first-line salvage therapy	yes	123	25.12	18.25-31.99	1.00		
	no	48	9.041	3.76-14.32	2.196	1.531-3.152	<0.001
Remission status	≥PR	150	22.92	19.40-26.43	1.00		
	<PR	21	6.25	4.28-8.21	3.492	2.165-5.633	<0.001
Conditioning	HD-Mel	154	21.30	17.12-26.49	1.00		
	BU-Mel	13	14.17	10.66-17.68	1.571	0.883-2.796	0.124
Pretreatment	ChT	53	19.86	13.35-26.37	1.00		
	≥1 novel drug	118	21.30	16.40-26.21	0.883	0.617-1.265	0.499
Salvage therapy	1 novel drug	80	20.75	17.52-23.97	1.00		
	>1 novel drug	89	19.10	12.05-26.15	1.048	0.742-1.481	0.790
Pretreatment regimens	≤2	132	21.30	16.92-25.68	1.00		
	>2	39	16.73	8.95-24.52	1.396	0.948-2.057	0.091
Lenalidomide maintenance therapy	no	151	20.65	14.43-26.86	1.00		
	yes	19	19.10	14.08-24.12	1.355	0.785-2.339	0.275
Time of Re-AHCT	2002-2013	71	14.17	11.11-17.23	1.00		
	2014-2021	100	24.36	18.79-29-93	0.679	0.481-0.960-	0.028

(b) – OS

Variable	Factor	n	Median OS (mo)	95% CI	HR	95% CI	*P* value
Sex	male	112	72.07	57.38-86.75	1.00		
	female	59	61.09	46.85-75.32	1.066	0.699-1.624	0.767
Age	≤65 years	105	60.23	39.39-81.14	1.00		
	>65 years	66	85.94	56.55-115.33	0.706	0.460-1.083	0.111
Previous AHCT	1	142	70.62	49.91-91.32	1.00		
	>1	29	61.09	49.30-72.87	1.397	0.878-2.221	0.158
Soft-tissue plasmacytoma	no	133	65.33	55.01-75.64	1.00		
	yes	38	72.07	35.33-108.8	1.301	0.797-2.126	0.293
Disease status	1 relapse	146	70.62	56.14-85.10	1.00		
	>1 relapse	25	53.39	27.61-79.18	1.489	0.861-2.573	0.154
DoR	>24 months	96	86.19	62.59-97.78	1.00		
	≤24 months	75	38.40	31.90-44.90	1.977	1.313-2.978	0.001
R-ISS	I	69	85.41	68.98-101.84	1.00		
	II + III	98	41.76	19.25-64.25	1.665	1.077-2.574	0.022
LDH	normal	91	80.19	59.75-100.62	1.00		
	elevated	69	56.84	28.78-84.91	1.404	0.916-2.152	0.120
HRCA (R-ISS)	no	116	80.19	61.75-98.63	1.00		
	yes	21	34.39	23.35-45.43	1.487	0.798-2.770	0.211
HRCA (R-ISS) and/or t(14;20) and/or gain/amp1q21	no	46	86.53	50.51-122.56	1.00		
	yes	52	62.43	29.48-95.39	1.402	0.777-2.529	0.262
Paraprotein type	others	128	71.70	60.26-83.14	1.00		
	IgA	43	56.84	43.47-70.22	1.247	0.782-1.988	0.354
Response to first-line salvage therapy	yes	123	80.19	61.31-99.06	1.00		
	no	48	34.46	17.87-51.04	1.984	1.311-3.005	0.001
Remission status	≥PR	150	80.19	63.67-96.71	1.00		
	<PR	21	13.64	0-37.04	3.880	2.371-6.351	<0.001
Conditioning	HD-Mel	154	71.70	54.73-88.68	1.00		
	BU-Mel	13	61.09	26.77-95.40	1.415	0.752-2.661	0.281
Pretreatment	ChT	53	55.04	32.56-77.51	1.00		
	novel drugs	118	76.04	55.37-96.72	0.747	0.489-1.139	0.175
Salvage therapy	1 novel drug	80	65.33	49.47-54.54	1.00		
	>1 novel drug	89	76.04	54.54-97.55	1.023	0.675-1.549	0.915
Pretreatment regimens	≤2	136	70.62	60.67-80.57	1.00		
	>2	35	53.39	28.16-78.63	1.138	0.716-1.810	0.585
Lenalidomide maintenance therapy	no	151	65.33	51.99-78.66	1.00		
	yes	19	63.42	33.71-93.13	0.990	0.477-2.056	0.979
Time of Re-AHCT	2002-2013	71	50.24		1.00		
	2014-2021	100	76.04	59.00-93.09	0.638	0.415-0.981	0.041

Table S2 Hazard ratios for progression-free survival (HR_{PFS}) and overall survival (HR_{OS}) using multivariate Cox regression analysis. All variables with *P*<0.1 in univariate analysis were included in the complete model for both, PFS and OS.

**Variable**	**HR_{PFS}**	**95%-CI**	***P* value**	**HR_{OS}**	**95%-CI**	***P* value**
Median age >65 years	0.734	0.490-1.098	0.132	-	-	-
Soft-tissue plasmacytoma	1.878	1.192-2.958	0.007	--	-	-
Disease status >1 relapse	1.251	0.733-2.136	0.411	-	-	-
DoR ≤24 months	1.799	1.234-2.624	0.002	1.900	1.230-2.933	0.004
R-ISS stage II + III	1.478	0.983-2.223	0.061	1.531	0.985-2.383	0.059
Paraprotein not IgA	1.306	0.833-2.049	0.244	-	-	-
No response to first-line salvage therapy	1.267	0.794-2.020	0.321	1.162	0.652-2.072	0.610
Remission status <PR	2.748	1.460-5.172	0.002	3.019	1.512-6.029	0.002
Pretreatment >2 regimens	1.588	0.985-2.558	0.058	-	-	-
Re-AHCT between 2002-2013	1.088	0.742-1.570	0.688	1.033	0.651-1.638	0.891
